# Long-Term Alterations of Glucocorticoid Receptor Expression and CD4^+^ T Cells in Adolescent Rhesus Macaques Following Early-Life Adversity

**DOI:** 10.3390/biom15121701

**Published:** 2025-12-05

**Authors:** Mar M. Sanchez, Leonidas Panagiotakopoulos, Timothy Hayes, Brittany R. Howell, Kelly Ethun, Kirk A. Easley, Guido Silvestri, Diane G. Carnathan, Jackson McCandless, Jerrold Meyer, Gretchen N. Neigh

**Affiliations:** 1 Department of Psychiatry & Behavioral Sciences, School of Medicine, Emory University, Atlanta, GA 30345, USA; mmsanch@emory.edu; 2 Department of Developmental and Cognitive Neuroscience, Emory National Primate Research Center, Emory University, Atlanta, GA 30345, USA; brhowell@vtc.vt.edu (B.R.H.); kelly.f.ethun@emory.edu (K.E.); 3 Department of Pediatrics, Division of Pediatric Endocrinology, School of Medicine, Emory University, Atlanta, GA 30345, USA; 4 Division of Microbiology and Immunology, Emory National Primate Research Center, Emory University, Atlanta, GA 30345, USAgsilves@emory.edu (G.S.); diane.g.carnathan@emory.edu (D.G.C.); 5 Fralin Biomedical Research Institute at Virginia Tech Carilion, Roanoke, VA 24061, USA; 6 Department of Human Development and Family Science, Virginia Tech, Blacksburg, VA 24061, USA; 7 Department of Biostatistics and Bioinformatics, Rollins School of Public Health, Emory University, Atlanta, GA 30322, USA; keasle2@emory.edu; 8 Department of Biological Sciences, Virginia Tech, Blacksburg, VA 24061, USA; 9 Department of Psychological and Brain Sciences, University of Massachusetts Amherst, Amherst, MA 01003, USA; jmeyer@umass.edu; 10 Department of Neuroscience & Anatomy, School of Medicine, Virginia Commonwealth University, 1101 East Marshall Street, Richmond, VA 23298, USA

**Keywords:** early-life adversity, nonhuman primate, FKBP5, glucocorticoid receptor, TNFα, CD4^+^, hair cortisol, chronic stress

## Abstract

Child maltreatment (MALT) is a devastating form of early-life adversity (ELA) and a primary risk for mental and physical illness. It is difficult to disentangle postnatal caregiving effects from heritable factors. Here we investigated the long-term effects of maternal care using a cross-fostering design to control for biological/heritable factors on immune function and inflammation during adolescence in a translational and naturalistic macaque model of MALT. We studied the impact of MALT on the immunophenotype of peripheral blood mononuclear cells (PBMCs) and assessed glucocorticoid receptor expression and function during adolescence. MALT was associated with elevated expression of *NR3C1*, the gene that encodes for the glucocorticoid receptor, in PBMCs. Glucocorticoid receptor function was not altered by MALT when examined for response to dexamethasone (DEX). In addition, MALT led to a reduction in the percentage of naïve CD4^+^ T cells and an increase in the percentage of central memory (Tcm) CD4^+^ T cells. These results suggest that MALT-exposed adolescents show residual effects of MALT on CD4^+^ T cells and increased expression of *NR3C1* without demonstration of increased function of the glucocorticoid receptor. Taken together, these results suggest that ELA has enduring implications for cellular glucocorticoid receptor biology and CD4^+^ T cells.

## 1. Introduction

Child maltreatment is a long-standing societal problem that is prevalent across all socioeconomic groups and present in multiple geographical areas and cultures [1,2,3]. It is a devastating form of early-life adversity (ELA) linked to an increasing number of child, adolescent, and adult physical and mental illnesses, including alterations in immune function and inflammation [4,5,6,7,8,9]. Despite these strong links, we still lack understanding of the underlying biological mechanisms and how these alterations unfold during development. ELA can impact the development of physiological stress systems, including the hypothalamic–pituitary–adrenal (HPA) axis, which has been shown to be directly affected by child maltreatment, both in the short and long term, in human and nonhuman primate (NHP) species [10,11,12]. A common outcome across many studies is the short-term over-reactivity of the HPA axis in subjects that have experienced early-life maltreatment, with higher baseline cortisol values [11,12,13] and increased responses to HPA axis stimulation [14]. Moreover, the effects of early childhood maltreatment appear to be long-lasting and affect the HPA axis in juveniles, adolescents, and adults [12,15,16,17]. Specifically, adults with a history of childhood maltreatment show hyperreactivity of the HPA axis [18,19,20] and have lower basal cortisol levels [21,22,23]. Work in animal models has demonstrated that ELA also affects the HPA axis at the subcellular level, with decreases in glucocorticoid receptor (GR) production in stress-sensitive areas of the CNS [24,25] and epigenetic changes on the GR gene detectable in adolescence [26,27]. These changes can affect glucocorticoid-mediated negative feedback on HPA axis activity and alter cellular effects of GR activation.

A translational model of childhood maltreatment has been reported in cercopithecinae monkeys living in large captive groups, where there is 2–5% incidence of infant physical abuse comorbid with neglect by rhesus monkey mothers [28,29,30]. Infant maltreatment (MALT) in rhesus monkeys happens in the first 3 months of life, equivalent to 1 year in humans [29], is repeated with all offspring [31], and shows transgenerational transmission along the maternal line [28], even in cross-fostering experiments [32]. Physical abuse and rejection cause infant distress and elevations of stress hormones [11,28]. Consistent with long-term impairments in stress and emotional regulation in children [12], our group has reported that infant MALT in rhesus monkeys also leads to long-term increased emotional reactivity, anxiety behaviors, hyperactivity of the HPA axis during infancy and the juvenile periods [11,12], and alterations in glucocorticoid negative feedback [12]. The effects of maltreating their infants (MALT) on the HPA axis last up to the third year of life, equivalent to 12 years in humans [11,33], but HPA axis activity normalizes by late adolescence (4.5–5.5 years of age), equivalent to 18–21 years in humans [12]. In a separate study evaluating the effects of sex and MALT on the HPA axis responses to pharmacological challenges, we showed that MALT animals had greater cortisol, but blunted adrenocorticotropic hormone (ACTH), responses to corticotropin-releasing factor (CRF) pharmacological stimulation. This effect was observed in the first few months of life and persisted until 3 years of age. The blunted ACTH levels in response to the CRF stimulation test in MALT compared to competent mothers (CON) were most prominent at 6 months of life, 3 months after the cessation of abusive behaviors [33]. These findings were interpreted as consistent with a hyperreactive HPA axis (greater cortisol responses to CRF challenge) in parallel to potential alterations in HPA axis glucocorticoid negative feedback (blunted ACTH responses to CRF) in MALT animals. In order to further understand the impact of MALT on GR biology at the cellular level, query of the molecular/cellular mechanisms underlying observed alterations in the HPA axis is required for interpreting the reported effects of MALT on HPA axis function. In addition to examination of the GR, insight can be gained through examination of FKBP5, a cochaperone of the GR that modulates the function of GR as a transcription factor [34,35,36,37]. A study by our group has shown an inverse relationship between FKBP5 DNA methylation and cortisol in infant monkeys born to maltreating mothers [38] and studies in rats have linked FBKP5 and GR levels in adulthood to ELA [39,40,41,42] and demonstrated reduced interactions between FBKP5 and GR following ELA [43]. Therefore, we tested the hypothesis that exposure to MALT alters *NR3C1* expression and responsivity when assessed *ex vivo* from samples of peripheral blood mononuclear cells (PBMCs) extracted from rhesus macaques with a history of MALT. We used a randomized cross-fostering experimental design where we assigned infants at birth to either mothers with a MALT or CON, in order to examine postnatal caregiving effects while accounting for confounding effects of heritable factors.

In addition to the impact of ELA on GR biology and HPA axis function, ELA has both direct and indirect effects on immune function and inflammation [7,8,9,44]. Early-life environmental toxin exposure has been documented to cause enduring changes in the CD4^+^ T cell transcriptome [45]. Furthermore, in adults over the age of 50 years, a history of early-life trauma and chronic stress has been associated with a reduced percentage of naïve CD4^+^ T cells and an increase in terminally differentiated CD4^+^ cells, suggesting accelerated immune aging [46]. The impact of ELA on inflammation and immune cell subsets has been documented as early in the life course as adolescence, including an increase in effector memory CD4^+^ T cells [47]. Effects of stress on immunophenotype have also been reported in adult rhesus macaques including experiences of social adversity in the form of low social status [48,49]. Furthermore, glucocorticoids, in particular their actions via binding to the GR, are poised to influence both T cell development and differentiation. Glucocorticoids influence T cells in multiple manners including programming of the T cell receptor repertoire in early life, influences of circadian fluctuations in glucocorticoids on T cell trafficking and responsiveness, and elevated glucocorticoid levels (above those typical to the peak of the circadian cycle) influence effector and memory T cell differentiation and responses. To this end, we also tested the hypothesis that a history of MALT would inhibit GR-induced suppression of an *ex vivo* inflammatory response and alter the T cell profiles of macaques with a history of MALT in the circulating pool of PBMCs.

## 2. Methods

### 2.1. Subjects and Housing

A total of 23 rhesus macaques (*M. mulatta*) were studied during early adolescence (ranging 1.9–4.8 years of age; mean 3.2, SD = 0.9 years) as part of a larger project investigating the developmental consequences of infant MALT [11,12]. MALT groups were exposed to physical abuse operationally defined as violent behaviors of the mother towards the infant, which caused pain and distress (dragging, crushing, throwing, stepping or sitting on, and rough grooming of the infant); these behaviors were comorbid with early infant rejection, operationalized as the mother preventing contact or infant access to her ventrum by pushing the infant away, blocking it with her arm or twisting her torso away, also causing infant distress [11,33].

Of the 23 subjects, 12 experienced maternal MALT (8 males, 4 females) and 11 were raised by competent mothers (CON: 4 males, 7 females). Extensive details of the maternal care received by these animals as infants (including the rates and duration of physical abuse and rejection) have been previously published [11]. To control for confounding effects of heritable/prenatal factors, we used a cross-fostering experimental design with random assignment of infants at birth to either CON or MALT foster mothers following published procedures for this cohort of animals [11]. Of the 11 infants raised by CON foster mothers, 7 were born to biological CON mothers (4 males, 3 females), and 4 to biological MALT mothers (4 females). Of the 12 infants raised by MALT foster mothers, 6 were born to biological CON mothers (5 males, 1 female) and 6 to other biological MALT mothers (3 males, 3 females). Analyses for this report are based on the foster mother (CON (*n* = 11) and foster MALT (*n* = 12)) with biological mother types represented in both groups.

Subjects lived with their mothers and families in large social groups with established matrilineal social hierarchies and 2–3 adult breeder males at the Emory National Primate Research Center (ENPRC) Field Station, Emory University (Lawrenceville, GA, USA). This high social complexity allowed us to balance the distribution of social dominance ranks (high, medium, low) in addition to sex and genetic diversity across groups. We excluded infants with birth weight < 450 g (3 throughout the larger study) because it is a clinical cut-off for prematurity in rhesus macaques, and birth weight is a strong predictor of biobehavioral development in primates [50]. The groups were housed in outdoor compounds with access to climate-controlled indoor areas. Standard high-fiber, low-fat monkey chow (Purina Mills LLC, St. Louis, MO, USA) and fruits and vegetables were provided twice daily, and water was available *ad libitum*. All procedures were performed in accordance with the Animal Welfare Act and the U.S. Department of Health and Human Services “Guide for the Care and Use of Laboratory Animals” and approved by the Emory Institutional Animal Care and Use Committee (IACUC).

### 2.2. Collection of Blood and Hair

Juvenile and adolescent macaques are independent of their mothers. Using positive reinforcement, subjects were trained to separate from the social group, move into an indoor room and then into a cage with a squeeze mechanism, where they were trained to present a leg for anesthesia (5 mg telazol/kg BW, i.m.) to collect blood samples from the femoral vein and hair for cortisol concentrations. Blood samples were collected within 10 min of disturbance to reflect baseline levels of physiological markers, as previously demonstrated [11,33]. Hair was shaved from the nuchal area (approx. 1 square inch) under anesthesia to measure prolonged cortisol accumulation. Blood samples (2 × 8 mL) were drawn from the femoral vein and placed in EDTA tubes stored on ice for processing for PBMC extraction through density centrifugation.

### 2.3. Hair Cortisol Assay

Hair cortisol accumulation has been used as a biomarker of integrated HPA axis activity over weeks and months, because it is constantly deposited in the growing hair shaft. This measurement has been used by many experimental laboratories to evaluate chronic stress-related cortisol production in humans, macaque monkeys, and other non-primate mammals [51]. Hair samples were processed and assayed as previously described [51]. Briefly, each sample was weighed, washed twice in isopropanol to remove external contamination, ground to a fine powder, and then extracted with methanol overnight. The methanol was evaporated, the residue was redissolved in assay buffer, and then the cortisol was measured using the Salimetrics (Carlsbad, CA, USA) enzyme immunoassay kit (cat. # 1-3002) according to the manufacturer’s directions. Intra- and inter-assay coefficients of variation were <10%.

### 2.4. PBMC Culture and Stimulation

PBMCs were isolated by gradient centrifugation as previously described [52,53]. The *ex vivo* nature of our study allowed for control of common confounding factors that interfere with social stress experiments, such as stress resulting from animal capture and handling [33]. A widely used proxy *in vitro* for stress-related levels of cortisol is cell incubation with dexamethasone (DEX) at 10^−8^ M for less than 24 h [54]. In addition, PBMCs are commonly stimulated with lipopolysaccharide (LPS) extract to induce an immunogenic response as early as the first few hours of exposure [55].

PBMCs were plated at a concentration of 0.5 × 10^6^ per well, and cultured in RPMI 1640 culture medium (Life Technologies, Carlsbad, CA, USA) supplemented with 5% heat-inactivated fetal bovine serum (Invitrogen, Grand Island, NY, USA), 1% penicillin/streptomycin (Invitrogen, Grand Island, NY, USA), and 1% L-Glutamine (Life Technologies, Carlsbad, CA, USA) at 37 °C in 5% CO_2_ in a humidified incubator [56]. At time = 0 h, DEX (Sigma Aldrich, St. Louis, MO, USA) was added at 10^−8^ M; at time = 6 h, LPS (Sigma Aldrich, St. Louis, MO, USA) was added at 0.1 μg mL^−1^. Samples were collected at time = 12 h from control, DEX, LPS, and DEX/LPS conditions.

### 2.5. Quantitative RT-PCR

Following PBMC collection from cultures, culture supernatant was immediately removed, and pelleted cells were rapidly frozen on dry ice. RNA was extracted with the TRIzol method (Invitrogen, Carlsbad, CA, USA) using an RNeasy Mini kit (Qiagen, Valencia, CA, USA). RNA concentration and integrity were assessed by a NanoDrop 2000 spectrophotometer (ThermoScientific, Wilmington, DE, USA). RNA was standardized to 4 ng/μL and reverse-transcribed with the High-Capacity RNA to cDNA kit (Applied Biosystems, Foster City, CA, USA). cDNA was quantified with the PicoGreen Assay (Invitrogen, Carlsbad, CA, USA) and then standardized so that all samples started quantitative RT-PCR with 5 pg of cDNA. The selection of an ideal endogenous control is important to the interpretation of real-time PCR experiments [57]. All DNA primers were designed using the Primer3Plus online software (https://www.primer3plus.com) [58] (see Table S1).

The primers were ordered from and manufactured by Integrated DNA Technologies (San Diego, CA, USA). Primers were validated using cDNA concentrations decreasing by a factor of 5 to the standard of −3.3 for the slope of the association between threshold cycle and log cDNA concentration [59]. qRT-PCR was performed using Absolute Blue qPCR SYBER Green ROM Mix (Thermo Scientific, Wilmington, DE, USA). The universal two-step RT-PCR cycling conditions used on the 7900HT Sequence Detection System (Applied Biosystems, Foster City, CA, USA) were 50 °C (2 min), 95 °C (10 min), 40 cycles of 95 °C (15 s) and 60 °C (1 min), then 95 °C (15 s), 60 °C (15 s). The expression of an endogenous reference gene, *TBP*, was confirmed to be stable among groups and culture conditions. Relative gene expression of individual samples run in duplicate (with coefficient of variation cut-off set to ≤4%) was calculated by the comparative Cycle Threshold quantification method relative to the change in the endogenous reference gene (dCT), as described previously [60].

### 2.6. TNFa Assay

Following cell culture harvest, cell media were spun down at 1000× *g* for 5 min. Supernatant was collected for the TNFα assay. Supernatant TNFα levels were assessed via ELISA: TNFα (sensitivity: 2.6–6.2 pg/mL, R&D systems, Minneapolis, MN, USA). Samples were run in duplicate.

### 2.7. Immunophenotyping and Flow Cytometry

Multi-parameter flow cytometric analysis was performed on mononuclear cells isolated from blood according to standard procedures [52,53]. Pre-determined optimal concentrations of antibodies listed in Table S2 were used. Flow cytometric acquisition was perfomred on an LSRII flow cytometer with the DiVa software package (version 6.1.3; BD Biosciences, San Jose, CA, USA). Analysis of the acquired data was performed using FlowJo software (version 9.4.4; Tree Star, Inc., Ashland, OR, USA). CD4^+^ T cell subsets were the focus of our hypotheses. Descriptive statistics for all cell subsets are provided in the Supplementary Materials.

### 2.8. Data Analysis

A mixed-effects model was used for the analysis of study endpoints with repeated measures (gene expression levels of *NR3C1*, *TNFα*, and *FKBP5*). The analyses were performed via the SAS MIXED Procedure (version 9.4; SAS Institute, Cary, NC, USA), providing separate estimates of the mean endpoint by foster MALT-related group (CON and MALT) and culture conditions (for *NR3C1* and *FKBP5*: baseline and after *ex vivo* culture with DEX; for *TNFα*: baseline and after *ex vivo* culture with DEX, LPS, and DEX + LPS). The statistical models included three predictors [MALT-related groups, culture conditions (categorical) and the statistical interaction between MALT-related group and culture conditions]. A compound-symmetric variance–covariance form in repeated measurements due to culture conditions was assumed for each endpoint, and robust estimates of the standard errors of parameters were used to perform statistical tests and construct 95% confidence intervals (CIs) [61]. The model-based means were unbiased with unbalanced and missing data, when the missing data were non-informative (missing at random). A *p*-value ≤ 0.05 was considered statistically significant for the main effects (MALT-related group and culture conditions) and for the MALT-related group by culture condition interaction effects. The statistical test for interaction between experimental group and culture conditions was an overall test to determine whether each endpoint in the MALT-related groups changed differentially across culture conditions (i.e., different patterns of change across culture conditions for the abuse-related study groups). If the mean endpoint in the study groups was similar over culture conditions (i.e., no statistical interaction), then the main effect test for abuse-related study groups was used as the primary hypothesis test to compare the study groups. The primary study results from this model were the mean endpoint, and the standard error of the mean for each of the abuse-related study groups plus the mean differences. Only main effects were detected and only two levels existed in these comparisons; therefore, pairwise posthoc assessments were not conducted. Cohen’s d is presented as a metric of effect size for mixed model analyses along with Intraclass Correlation Coefficient (ICC). Descriptive statistics for *NR3C1*, *FKBP5*, and *TNFα* expression are provided in Tables S3–S5.

For outcomes with a single endpoint per subject (hair cortisol, TNFα DEX suppression, and flow cytometry endpoints), two-tailed unpaired *t*-tests with Welch’s correction were used to compare outcomes between foster CON and MALT groups. The Welch correction was selected because it is conservative, does not assume equal variance among groups, and does not require equal sample sizes [62]. These statistical analyses were performed using GraphPad Prism v.10.4.2 (La Jolla, CA, USA). Group means were determined to be statistically significant when *p* < 0.05, and are presented as a mean ± 95% CI. Cohen’s d is presented as a metric of effect size for analyses that utilized two-tailed unpaired *t*-tests. Statistical analysis of immunophenotype was limited to CD4^+^ T cells (%, naïve, Tcm, Tem) from PBMCs. Descriptive statistics for these, and all other immunophenotype endpoints examined, are presented in Tables S6–S9.

## 3. Results

### 3.1. MALT Did Not Alter Hair Cortisol

**Figure 1:**
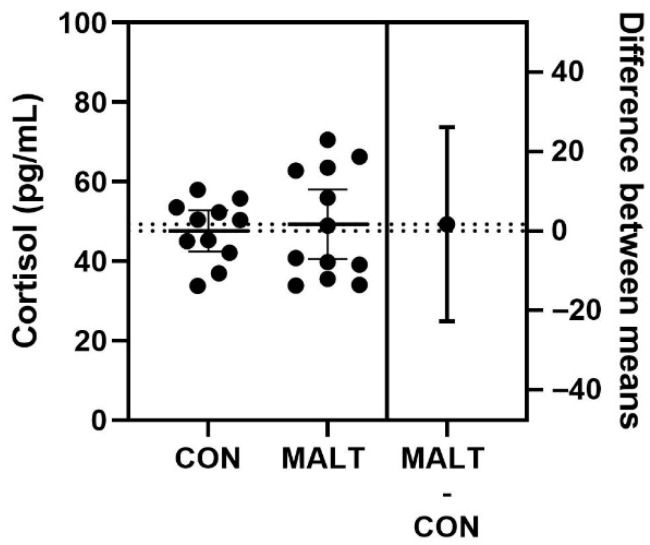
Impact of MALT on hair cortisol. Cortisol concentrations in hair samples obtained from offspring reared by foster mothers with demonstration of competent maternal behaviors (CON) or that exhibited maltreatment of their infant (MALT) did not differ. Individual data points are illustrated on the graph. The difference between the means and 95% CI is represented on the right *y*-axis.

Hair cortisol (Figure 1) was similar between the CON and MALT groups (t_21_ = 0.35, *p* = 0.73, *d* = 0.09).

### 3.2. MALT Rearing Altered Expression of NR3C1 in PBMCs

**Figure 2:**
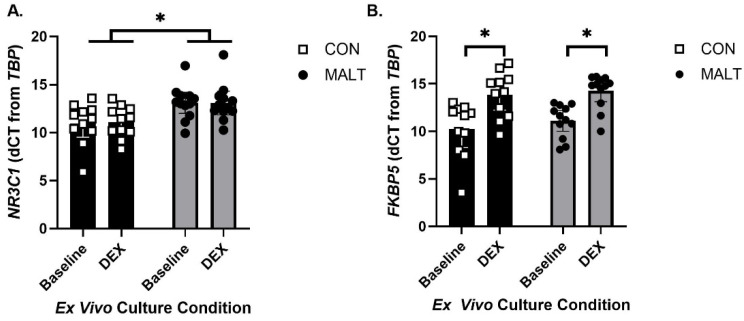
Gene expression levels for *NR3C1* and *FKBP5* by MALT and DEX. *NR3C1* (**A**) and *FKBP5* (**B**) expression in CON foster conditions (black bars), and in animals with a history of MALT exposure (gray bars). Gene expression is normalized to *TBP*. (**A**) Expression of *NR3C1* was elevated in subjects with a history of MALT and this was not altered by exposure of the cells to dexamethasone (DEX). (**B**) Gene expression for *FKBP5* was not altered by MALT. PBMCs from all offspring contained functional glucocorticoid receptors such that both cells from both CON and MALT subject manifested an increase in *FKBP5* expression following DEX stimulation. All data are represented as mean ± 95% CI, and the symbol * indicates a statistically significant difference between annotated groups (*p* < 0.05).

A mixed-effects model assessment of expression of the GR gene (gene: *NR3C1*; Figure 2A) provided separate estimates of the mean endpoint by MALT-related group (CON, MALT) and culture conditions (baseline and after ex vivo culture with DEX). The model demonstrated that *NR3C1* expression differed as a function of MALT (*p* = 0.01, *d* = 1.12, ICC = 0.91) but not in response to culture condition (*p* = 0.69). MALT and culture condition did not interact to influence expression of *NR3C1* (*p* = 0.63).

A mixed-effects model assessment of expression of *FKBP5* in PBMCs (Figure 2B) provided separate estimates for each rearing group and *ex vivo* culture condition. The model demonstrated that *FKBP5* expression was not impacted by MALT (*p* = 0.47). PBMCs from all groups were responsive to DEX stimulation such that DEX increased expression of *FKBP5* (*p* < 0.0001, *d* = 1.61, ICC = 0.91). There was no interaction between MALT and culture condition (*p* = 0.25).

### 3.3. Ex Vivo TNFα Gene Expression and DEX Suppression of TNFα Protein Was Not Altered by MALT

**Figure 3:**
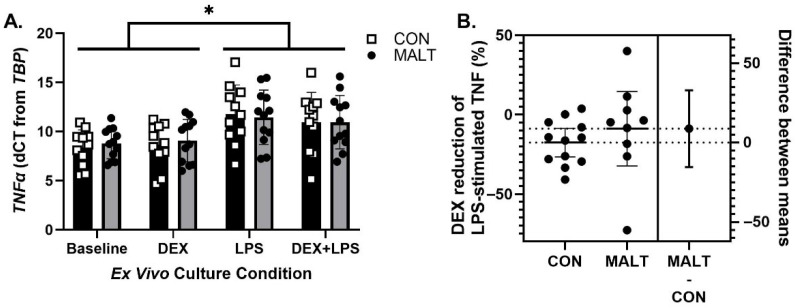
*TNFα* gene expression levels and dexamethasone (DEX) suppression. Changes in *TNFα* (**A**) and DEX suppression of TNFα (**B**). Expression of *TNFα* was altered by exposure of the PBMCs to lipopolysaccharide (LPS). There was no impact of MALT on the ability of DEX to alter the production of TNFα in response to LPS. Data are represented as mean ± 95% CI and as individual data points and 95% CI of the mean difference. The figure symbol (*) indicates a statistically significant difference between annotated groups (*p* < 0.05).

A mixed-effects model assessment of expression of *TNFα* (Figure 3A) demonstrated that expression of *TNFα* was not altered by MALT (*p* = 0.88), but was responsive to *ex vivo* stimulation with LPS (*p* < 0.0001, *d* = 1.26, ICC = 0.77). MALT and culture condition did not interact to influence expression of *TNFα* (*p* = 0.63).

TNFα protein concentrations were measured after *ex vivo* culture with LPS and DEX + LPS in order to calculate percent DEX suppression (Figure 3B). A history of MALT did not alter the ability of DEX to suppress LPS-stimulated production of TNFα (t_10.55_ = 0.80, *p* = 0.44, *d* = 0.38).

### 3.4. Immunophenotype of CD4^+^ T Cells Within PBMCs Was Altered by MALT Rearing

**Figure 4:**
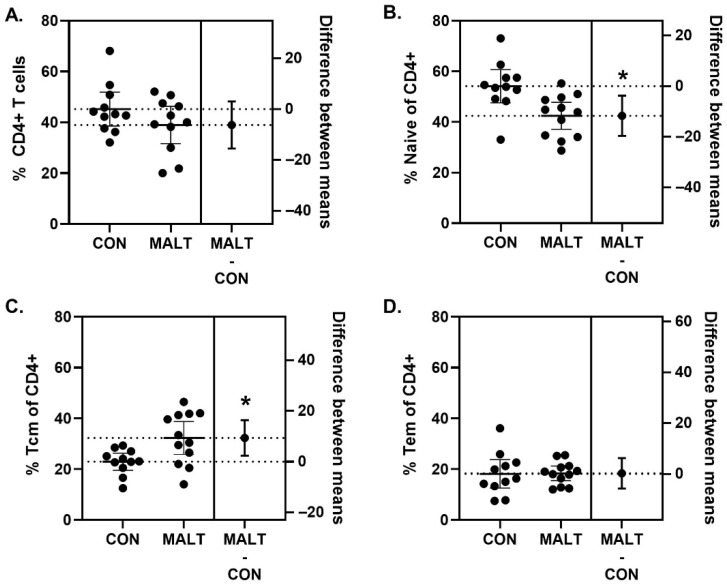
MALT alters CD4^+^ T cells. Although the percentage of cells identified as CD4^+^ cells did not differ because of MALT (**A**), rearing by a MALT mother reduced the percentage of naïve CD4^+^ T cells (**B**). The percentage of CD4^+^ T cells that were identified as Tcm cells was elevated in offspring reared by MALT foster mothers (**C**). MALT did not alter the percentage of Tem CD4^+^ T cells (**D**). All data are represented as individual data points with a line indicating the mean. The difference in means and 95% CIs is indicated on the right *y*-axis. The figure symbol (*) indicates a statistically significant difference between groups (*p* < 0.05).

There was no difference in percentage of CD4^+^ T cells among PBMCs from CON and MALT groups (t_19.81_ = 1.42, *p* = 0.17, *d* = 0.57) (Figure 4A; Table S6). MALT reduced the percentage of CD4^+^ T cells that were classified as naïve (t_19.80_ = 3.06, *p* = 0.006, *d* = 1.31). CD4^+^ T cells classified as central memory T cells (Tcm) were higher in the MALT group than in the CON group (t_16.27_ = 2.83, *p* = 0.01, *d* = 1.14; Figure 4C). CD4^+^ T cells classified as effector memory T cells (Tem) did not differ based on foster group (t_15.15_ = 0.07, *p* = 0.95, *d* = 0.00; Figure 4D).

## 4. Discussion

The current body of work assessed whether ELA, in the form of maternal maltreatment during infancy, has long-lasting effects in early adolescence in rhesus monkeys (3 years of age, equivalent to 12 years in humans) with respect to the expression and function of the GR and immunophenotype. Collectively, the data presented here demonstrate that MALT experience in nonhuman primates leads to increased expression of *NR3C1*, the gene that encodes GR, without demonstration of enhanced GR function. This could suggest that molecular-level changes in GR biology, as a function of MALT, may be compensating for reduced function of the GR, or HPA axis perturbations overall. In addition, assessment of T cell phenotype in PBMCs demonstrated a shift in CD4^+^ T cells such that there was a reduction in naïve CD4^+^ cells and an increase in Tcm CD4^+^ T cells for the MALT group, suggesting a potential acceleration of immune aging processes. While the dataset available in the current study was limited, the novel data presented demonstrate prolonged effects of MALT on both GR biology and CD4^+^ T cell immunophenotype, suggesting an important area of further study to develop an understanding of the effects of ELA on health and disease across the lifespan.

In addition to the important contributions of this study, there are also several limitations that should be discussed. First, although the sample size is consistent with the field for this type of macaque study [11,12], the study lacked statistical power to investigate complex interactions between some important variables, particularly for biological mother and sex. Although the foster care groups were counterbalanced to control for the potential effects of heritable and prenatal factors from the biological mother through the cross-fostering experimental design with randomized assignment to foster groups at birth, we were underpowered to examine foster by biological mother interaction effects for the outcomes reported. Future work should include larger sample sizes that can support assessment of interactions among MALT and the effects of biological mother and sex in order to build upon the results reported here that controlled for biological mother with the counterbalanced experimental design. In addition to the cross-fostering design employed in this study to control for heritable and prenatal confounds, the potential exposure to different levels of prenatal stress was ruled out by measuring hair cortisol accumulation at birth, which was not different in newborns from biological control or maltreating mothers [11]. Despite our careful experimental controls, we cannot rule out effects of other confounding factors, particularly exposure to different immune prenatal environments.

Our current knowledge of the effects of ELA on the subcellular components of GR, one of the main effectors of the HPA axis, is limited, especially when it comes to primate species. Recent studies on rodents have shown epigenetic changes at the end of ELA exposure in the hypothalamus, which affect the degree of *nr3c1* gene expression [27]. However, little is known about the lasting effects of ELA exposure. Our results showed a significant increase in *NR3C1* expression in cultured PBMCs for the animals that had been exposed to MALT early in life. Of note, *NR3C1* expression levels in PBMCs did not change markedly with exposure to DEX for 12 h. This is in accordance with *in vitro* studies where *NR3C1* expression has been shown not to be altered by corticosteroids in neuronal cells, and only decreased after 4 days of DEX exposure in hippocampal cells [63]. Importantly, these results show a sustained alteration of expression of *NR3C1* in peripheral cells after ELA exposure that is still present in adolescence. This change could reflect enhanced receptor sensitivity or compensatory up-regulation in response to GR resistance, but should be interpreted with caution given that we do not provide information on the levels of GR protein or translocation status, which are important for full understanding of changes in GR biology.

We previously reported in a longitudinal study of the impact of infant MALT on the development of HPA axis function that infant MALT led to hyperactivity of the HPA axis during infancy followed by normalization by adolescence [12]. Consistent with this previous report, here we report that hair cortisol accumulation was similar between CON and MALT groups measured during adolescence. Normalized cortisol by adolescence is consistent with evidence of normalization of HPA axis function during adolescence in children that switch from adverse/deprived environments into supportive adoptive families [64]. Despite HPA axis function normalization in the ELA group during adolescence, exposure to high basal cortisol levels for the first 12 months of life can lead to alterations in physiological, immune, and metabolic function. Thus, the combination of increased *NR3C1* expression in animals exposed to infant MALT, concurrent with similar amounts of cortisol production, may suggest that the former is due to increased resistance at the level of the GR signal propagation, and this will be an important hypothesis to assess in a future study focused on protein- and translocation-level indicators for GR. These changes potentially have occurred through programming effects of early exposure to high cortisol secretion from infancy through 12 months [11,12]. In accordance, ELA studies in humans have indicated relative glucocorticoid resistance [65]. Even though there is an abundance of data suggesting changes in GR level expression at the time of or soon after exposure to ELA [24], we have limited knowledge on the longevity of such alterations. Our study is the first to show sustained effects of ELA on peripheral *NR3C1* expression levels in rhesus macaques, and in doing so provides new insight into the possible chronic changes that ELA might have on the stress response.

As mentioned, a key player in the functional control of the GR within the cytoplasm is its negative co-chaperone FKBP5 [34]. The relationship between FKBP5, GR function, and stress phenotypes has been detailed in both human investigations and work with animal models. Studies have shown that acute and repeated stress exposure leads to a reduction in *fkbp5* in critical CNS areas involved in the HPA axis of rats [66], presumably in an attempt to promote glucocorticoid signaling in stressful situations. However, these changes occur concurrently with stress exposure, and little is known about the long-term effects of stress on FKBP5 modulation. In our study, *FKBP5* gene expression was not altered in MALT offspring. In addition, the expression of the *FKBP5* gene was still responsive to GR stimulation as shown by the increase in *FKBP5* after DEX exposure, in accordance with previous reports in the literature [67,68]. This observed change is in support of the ultra-short feedback loop in the GR machinery that is thought to exist intracellularly [69], whereby exposure to the ligand (glucocorticoid) increases the amount of the negative cytoplasmic regulator (FKBP5) of the receptor–ligand complex. These data suggest that GR-mediated transcription, at least of *FKBP5*, is intact in the PBMCs of MALT offspring.

We also assessed the function of the GR by examining the ability of stimulation of the GR to suppress LPS-induced changes in TNFα. We examined both gene expression and protein levels of TNFα. All PBMC samples, regardless of rearing condition, generated a similar response to LPS in terms of gene expression. When we considered the ability of the GR to suppress the inflammatory response induced by LPS stimulation *ex vivo*, we observed a similar group-level ability for DEX to suppress the LPS response. In alignment with the *FKBP5* data discussed above, these data suggest that although we observed increased expression of *NR3C1* which encodes for the GR, the impact of stimulating the GR with DEX was equivalent among groups. This may suggest that a greater presence of GRs is necessary to produce a similar effect. However, the endpoint for GR presence in this study was gene expression of *NR3C1*, and future work will be necessary to determine if the GR is present in greater quantities at the protein level and examine localization of the receptor in order to better understand the impact of infant MALT on long-term GR function in different tissues. In rats, it has been previously reported that ELA leads to increased interactions between GR and FKBP5 and concomitant decreases in GR translocation which could account for alterations in GR efficacy [43].

We also examined the impact of MALT on immunophenotype of PBMCs with a focus on CD4^+^ T cells. Glucocorticoids have multifaceted effects on T cell development, differentiation and function, and most T cells express GRs [70]. In addition, different forms of ELA have been shown to be transcriptionally embedded within the immune system in a cell subtype-specific manner, where the directionality of effects is different depending on the nature of the lymphocyte in question [71]. Given our finding that MALT increased expression of *NR3C1*, and previous reports of ELA impacts on CD4^+^ T cells [7], we queried the potential impact on T cell differentiation. MALT exposure was associated with a reduction in naïve CD4^+^ T cells and an increase in Tcm CD4^+^ T cells in PBMC samples. This is consistent with previous reports of trauma exposure on T cells with a pattern similar to aging with a reduction in naïve T cells and an enhancement in central memory cells [46,72]. These data align with the observation that early-life exposures are capable of generating enduring changes in the CD4^+^ T cell transcriptome [45] which have been linked to accelerated immune maturation/aging. The results reported highlight the importance of developing a better understanding of long-term effects of ELA on GR biology and shaping of immunophenotypes. Future studies should include characterization of immune responsivity and mechanisms that mediate the MALT-induced shift in the CD4^+^ T cell immunophenotype.

## 5. Conclusions and Future Directions

In this study, we demonstrated lasting effects of ELA on the intracellular components of the HPA axis and immunophenotypes. To date, this is the first study of its kind in rhesus macaques, and the first to focus on intracellular GR function in PBMCs. PBMC *NR3C1* transcript levels are elevated in adolescent rhesus macaques that have been previously exposed to MALT in infancy. Our study provides a foundation upon which a deeper characterization of the changes conferred by ELA onto the HPA axis is possible. Future studies should focus on understanding GR protein presence, GR nuclear translocation patterns, evaluation of GR-DNA binding patterns, and evaluation of GR-related gene targets. Furthermore, given the timing of the ELA in this study, focusing on epigenetic changes that involve the GR gene after exposure to forms of ELA in childhood [73] and in adolescence [74] could be particularly impactful. In addition, our study is the first to report distinct effects of maternal care on adolescent immunophenotypes, controlling for heritable/biological factors through a randomized cross-fostering study design. Taken together, these results suggest that ELA has enduring implications for expression of *NR3C1* and CD4^+^ T cell subtypes.

## Data Availability

The raw data supporting the conclusions of this article will be made available by the authors on request. Data collection for the animals in this manuscript is ongoing for adult endpoints.

## Supplementary Materials

The following supporting information can be downloaded at https://www.mdpi.com/article/10.3390/biom15121701/s1. Methods related to rectal biopsy flow cytometry; Table S1: Primer Specifications; Table S2: Antibodies for Flow Cytometry; Table S3: *NR3C1* PBMC Descriptive Statistics; Table S4: *FKBP5* PBMC Descriptive Statistics; Table S5: *TNFα* PBMC Descriptive Statistics; Table S6: CD4 PBMC Descriptive Statistics; Table S7: CD8 PBMC Descriptive Statistics; Table S8: CD4 Rectal Biopsy Descriptive Statistics; Table S9: CD8 Rectal Biopsy Descriptive Statistics.

## Abbreviations

The following abbreviations are used in this manuscript:

ELA	Early-life adversity
MALT	Maternal maltreatment
CON	Control
DEX	Dexamethasone
LPS	Lipopolysaccharide
GR	Glucocorticoid Receptor
NHP	Nonhuman Primate
CRF	Corticotropin-Releasing Factor
ACTH	Adrenocorticotropic Hormone
PBMC	Peripheral Blood Mononuclear Cell
HPA	Hypothalamic–Pituitary–Adrenal
ENPRC	Emory National Primate Research Center
CI	Confidence Interval
*d*	Cohen’s d
ICI	Intraclass Correlation Coefficient
Tem	Effector memory T cells
Tcm	Central memory T cells
