# Supplementary material for: Long-Term Alterations of Glucocorticoid Receptor Expression and CD4+ T Cells in Adolescent Rhesus Macaques Following Early-Life Adversity
Source: Biomolecules. 2025 Dec 5;15(12):1701. doi: 10.3390/biom15121701 (PMC12731112; doi:10.3390/biom15121701)
Supplement: Supplementary file 1 [file biomolecules-15-01701-s001.zip › biomolecules-3729384-supplementary.pdf]

Supplemental Methods for Rectal Biopsy Assessment

Immune cells exist both in circulation and localized to tissues throughout the body and early life adversity (ELA) has the capacity to impact both circulating and compartment-level immunophenotype. To this end, we also examined the T cell profiles of macaques with a history of MALT at the level of a tissue compartment (rectal biopsy).

Anesthesia and Sample Collection. As described in the main text, juvenile and adolescent macaques are independent of their mothers. Using positive reinforcement, subjects were trained to separate from the social group, move into an indoor room and then into a cage with a squeeze mechanism, where they were trained to present a leg for anesthesia (5mg telazol/kg BW, i.m.) to collect blood samples from the femoral vein (results in main manuscript), hair for cortisol concentrations (results in main manuscript) and rectal biopsies (reported in supplemental only). For rectal biopsies, following removal of fecal material from the rectum using a cotton applicator, an anoscope was placed a short distance into the rectum and 10 rectal pinches were obtained with biopsy forceps by an ENPRC veterinarian (KE). A subset of the biopsies collected were placed in tissue culture fluid for flow cytometry studies and outcomes reported in this manuscript.

Immunophenotyping and Flow Cytometry. Multi-parameter flow cytometric analysis was performed on mucosal tissues, i.e., rectal biopsies, as described in the main text. Pre-determined optimal concentrations of antibodies listed in Table S2 were used. Flow cytometric acquisition on an LSRII flow cytometer with the DiVa software package (from BD Biosciences, San Jose, CA). Analysis of the acquired data were performed using FlowJo software (Tree Star, Inc., Ashland, OR). Descriptive statistics for all cell subsets are provided in the Supplementary Materials.

Table S1. Primer specifications. FKBP5: FK506 Binding Protein 5; NR3C1: nuclear receptor subfamily 3, group C, member 1; TNFα: Tumor Necrosis Factor alpha; TBP: TATA-binding protein.

Gene	Forward Primer Sequence	Reverse Primer Sequence
NR3C1	CCAGCATGAGACCAGATGTA	CCACTGCTCTTTTGAAGAAA
FKBP5	CTTGCTGCCTTTCTGAACCT	CCCTTGGCTGACTCAAACTC
TNFα	CTGAGGTCAATCTGCCCAAGTAC	CTTCACAGAGCAATGACTCCAAAG
TBP	GTCCAGACTGGCAGCAAGAA	AAACTGTTGGTGGGTGAGCA

Table S2. Antibodies for Flow Cytometry

Antibody	Vendor
LIVE/DEAD aqua blue dead cell stain kit	Invitrogen, Carlsbad, CA
anti-CD8 BV711 (clone RPA-T8)	Biolegand, San Diego, CA
anti-HLA-DR APC-Cy7 (clone L243)	Biolegand, San Diego, CA
anti-CD184 (CXCR4) BV421 (clone: 12G5)	Biolegand, San Diego, CA
anti-CD4 BV650 (clone OKT4)	Biolegand, San Diego, CA
anti-CCR7 PE-Cy7 (clone 3D12)	BD Biosciences, San Jose, CA
anti-CCR5 PE (clone 3A9)	BD Biosciences, San Jose, CA
anti-CD95 PE-Cy5 (clone DX2)	BD Biosciences, San Jose, CA
anti-CD3 Alexa700 (clone SP34-2)	BD Biosciences, San Jose, CA
anti-integrin beta 7 (B7) APC (clone FIB504)	BD Biosciences, San Jose, CA
anti-Ki-67 FITC (clone B56)	BD Biosciences, San Jose, CA
anti-CD28 ECD (clone 28.2)	Beckman Coulter, Brea, CA
anti-CD279 (PD-1) PerCP-Cy5.5 (clone eBioJ105 (J105))	eBiosciences

Table S3. *NR3C1* PBMC Descriptive Statistics

	Biological	CONTROL	MALT	CONTROL	MALT
	Foster	CONTROL	CONTROL	MALT	MALT
Control	# of values	7	4	6	6
	Mean	11.61	9.83	12.97	13.24
	Std. Error of Mean	0.44	1.32	0.95	0.14
	Lower 95% CI of mean	10.73	7.17	11.08	12.96
	Upper 95% CI of mean	12.49	12.48	14.84	13.51
DEX	# of values	7	4	6	6
	Mean	11.51	10.37	13.17	13.01
	Std. Error of Mean	0.49	0.86	1.02	0.31
	Lower 95% CI of mean	10.53	8.66	11.13	12.39
	Upper 95% CI of mean	12.49	12.09	15.22	13.64
LPS	# of values	7	4	6	6
	Mean	12.28	10.79	13.03	13.43
	Std. Error of Mean	0.47	0.91	1	0.2
	Lower 95% CI of mean	11.33	8.98	11.03	13.03
	Upper 95% CI of mean	13.22	12.61	15.02	13.83
DEX + LPS	# of values	7	4	6	6
	Mean	11.99	10.66	13.14	13.65
	Std. Error of Mean	0.48	0.94	0.97	0.26
	Lower 95% CI of mean	11.04	8.77	11.21	13.13
	Upper 95% CI of mean	12.95	12.54	15.08	14.17

Table S4. *FKBP5* PBMC Descriptive Statistics

	Biological<br>Foster	CONTROL<br>CONTROL	MALT<br>CONTROL	CONTROL<br>MALT	MALT<br>MALT
Control	# of values	7	4	6	6
	Mean	11.11	8.66	10.04	12.18
	Std. Error of Mean	0.65	1.72	0.64	0.35
	Lower 95% CI of mean	9.82	5.23	8.75	11.47
	Upper 95% CI of mean	12.41	12.1	11.32	12.88
DEX	# of values	7	4	6	6
	Mean	14.58	12.59	13.36	15.19
	Std. Error of Mean	0.77	1.07	0.82	0.19
	Lower 95% CI of mean	13.03	10.45	11.71	14.81
	Upper 95% CI of mean	16.13	14.73	15.01	15.57
LPS	# of values	7	4	6	6
	Mean	11.81	9.75	10.26	12.27
	Std. Error of Mean	0.57	1.03	0.92	0.41
	Lower 95% CI of mean	10.67	7.68	8.42	11.45
	Upper 95% CI of mean	12.95	11.82	12.1	13.09
DEX + LPS	# of values	7	4	6	6
	Mean	14.24	12.42	13.12	14.79
	Std. Error of Mean	0.62	0.92	0.82	35
	Lower 95% CI of mean	13.01	10.58	11.48	14.09
	Upper 95% CI of mean	15.48	14.27	14.76	15.49

Table S5. *TNFα* PBMC Descriptive Statistics

	Biological<br>Foster	CONTROL<br>CONTROL	MALT<br>CONTROL	CONTROL<br>MALT	MALT<br>MALT
Control	# of values	7	4	6	6
	Mean	8.8	7.55	8.27	9.33
	Std. Error of Mean	0.55	0.97	0.64	0.48
	Lower 95% CI of mean	7.71	5.62	6.98	8.36
	Upper 95% CI of mean	9.9	9.49	9.55	10.3
DEX	# of values	7	4	6	6
	Mean	9.56	7.1	8.49	9.68
	Std. Error of Mean	0.42	1.22	0.85	0.77
	Lower 95% CI of mean	8.71	4.65	6.79	8.14
	Upper 95% CI of mean	10.41	9.55	10.2	11.21
LPS	# of values	7	4	6	6
	Mean	12.3	10.81	10.62	12.3
	Std. Error of Mean	0.67	1.96	1.03	1.02
	Lower 95% CI of mean	10.95	6.89	8.56	10.25
	Upper 95% CI of mean	13.65	14.73	12.68	14.35
DEX + LPS	# of values	7	4	6	6
	Mean	11.54	9.96	9.96	11.66
	Std. Error of Mean	0.63	2.03	2.03	0.93
	Lower 95% CI of mean	10.28	5.9	5.9	9.79
	Upper 95% CI of mean	12.79	14.01	14.01	13.53

Table S6. CD4 PBMC Descriptive Statistics

	Biological<br>Foster	CONTROL<br>CONTROL	MALT<br>CONTROL	CONTROL<br>MALT	MALT<br>MALT
%CD4+	# of values	7	4	6	6
	Minimum	36	32	30	20
	Maximum	68	46	52	48
	Range	32	14	22	28
	Mean	48	41	44	35
	Std. Deviation	11	6.2	8.2	11
	Std. Error of Mean	4.3	3.1	3.3	4.7
	Lower 95% CI of mean	37	31	35	23
	Upper 95% CI of mean	58	51	52	47
CD4+ Naïve	# of values	7	4	6	6
	Minimum	49	33	34	29
	Maximum	73	63	55	49
	Range	24	30	21	20
	Mean	57	49	47	38
	Std. Deviation	7.6	12	7.3	7.6
	Std. Error of Mean	2.9	6.2	3	3.1
	Lower 95% CI of mean	50	30	39	30
	Upper 95% CI of mean	64	69	54	46
CD4+ Tcm	# of values	7	4	6	6
	Minimum	13	21	21	14
	Maximum	29	29	41	47
	Range	17	8	21	33
	Mean	22	24	32	33
	Std. Deviation	5.8	3.4	7.9	13
	Std. Error of Mean	2.2	1.7	3.2	5.3
	Lower 95% CI of mean	17	19	24	19
	Upper 95% CI of mean	28	30	40	46
CD4+ Tem	# of values	7	4	6	6
	Minimum	7.8	7.5	12	12
	Maximum	26	36	25	26
	Range	18	29	13	14
	Mean	17	20	17	19
	Std. Deviation	6.1	12	4.7	4.5
	Std. Error of Mean	2.3	6.1	1.9	1.8
	Lower 95% CI of mean	11	0.65	12	15
	Upper 95% CI of mean	23	39	22	24
CD4+ B7	# of values	7	4	6	6
	Minimum	5.4	3	5	3.7
	Maximum	35	36	15	28
	Range	30	33	10	24
	Mean	15	15	9.7	11
	Std. Deviation	12	15	3.4	9
	Std. Error of Mean	4.5	7.3	1.4	3.7
	Lower 95% CI of mean	4.1	-8	6.2	2
	Upper 95% CI of mean	26	38	13	21

	Biological<br>Foster	CONTROL<br>CONTROL	MALT<br>CONTROL	CONTROL<br>MALT	MALT<br>MALT
CD4+ CCR5	# of values	7	4	6	6
	Minimum	3.7	6.6	6.3	8
	Maximum	20	30	33	37
	Range	17	23	27	29
	Mean	13	16	21	18
	Std. Deviation	6.6	12	10	11
	Std. Error of Mean	2.5	5.8	4.2	4.4
	Lower 95% CI of mean	7.1	-1.9	10	7.2
	Upper 95% CI of mean	19	35	32	30
CD4+ CXCR4	# of values	7	4	6	6
	Minimum	48	15	34	20
	Maximum	71	71	79	80
	Range	24	56	45	60
	Mean	59	46	65	52
	Std. Deviation	9	29	17	22
	Std. Error of Mean	3.4	14	6.9	9.1
	Lower 95% CI of mean	51	-0.24	47	28
	Upper 95% CI of mean	67	91	83	75
CD4+ HLA DR	# of values	7	4	6	6
	Minimum	0.93	2.1	7.5	1.2
	Maximum	14	14	22	19
	Range	13	12	14	18
	Mean	7.9	8	13	10
	Std. Deviation	4.6	5	5.2	7.5
	Std. Error of Mean	1.7	2.5	2.1	3.1
	Lower 95% CI of mean	3.6	0.12	7.3	2.4
	Upper 95% CI of mean	12	16	18	18
CD4+ Ki67	# of values	7	4	6	6
	Minimum	3.9	10	11	8.5
	Maximum	21	22	24	51
	Range	17	12	13	42
	Mean	12	17	15	21
	Std. Deviation	5.2	5.8	4.8	15
	Std. Error of Mean	2	2.9	1.9	6.2
	Lower 95% CI of mean	6.8	7.5	10	5.2
	Upper 95% CI of mean	16	26	20	37
CD4+ PD1	# of values	7	4	6	6
	Minimum	1.4	0.51	0.66	0.72
	Maximum	6.1	9.5	3.8	7
	Range	4.7	9	3.1	6.3
	Mean	3.6	3.2	2.8	2.9
	Std. Deviation	1.7	4.2	1.1	2.3
	Std. Error of Mean	0.64	2.1	0.45	0.95
	Lower 95% CI of mean	2	-3.5	1.7	0.5
	Upper 95% CI of mean	5.1	10	3.9	5.4

Table S7. CD8 PBMC Descriptive Statistics

	Biological	CONTROL	MALT	CONTROL	MALT
	Foster	CONTROL	CONTROL	MALT	MALT
	# of values	7	4	6	6
	Minimum	19	40	33	35
	Maximum	47	51	55	72
	Range	28	11	21	37
%CD8+	Mean	38	46	40	52
	Std. Deviation	10	4.5	7.9	15
	Std. Error of Mean	3.8	2.2	3.2	5.9
	Lower 95% CI of mean	29	38	32	37
	Upper 95% CI of mean	47	53	49	67
	# of values	7	4	6	6
	Minimum	5.9	7.8	10	2.7
	Maximum	30	47	26	39
	Range	24	39	15	36
CD8+ Naïve	Mean	21	25	19	13
	Std. Deviation	8.7	18	5.2	13
	Std. Error of Mean	3.3	9.2	2.1	5.4
	Lower 95% CI of mean	13	-3.9	13	-0.32
	Upper 95% CI of mean	29	54	24	27
	# of values	7	4	6	6
	Minimum	4.4	4.6	4.1	3.7
	Maximum	14	8.1	13	16
	Range	9.2	3.6	8.9	12
CD8+ Tcm	Mean	7.9	5.9	8.3	7.6
	Std. Deviation	3.4	1.6	3.3	4.3
	Std. Error of Mean	1.3	0.78	1.3	1.8
	Lower 95% CI of mean	4.8	3.4	4.9	3.1
	Upper 95% CI of mean	11	8.4	12	12
	# of values	7	4	6	6
	Minimum	49	40	63	44
	Maximum	87	83	83	89
	Range	38	43	20	45
CD8+ Tem	Mean	67	65	69	75
	Std. Deviation	13	19	7.4	17
	Std. Error of Mean	4.8	9.4	3	6.8
	Lower 95% CI of mean	55	35	61	58
	Upper 95% CI of mean	79	94	77	93
	# of values	7	4	6	6
	Minimum	16	13	9.4	14
	Maximum	32	15	33	29
	Range	16	1.9	23	15
CD8+ B7	Mean	23	14	21	22
	Std. Deviation	6.1	0.84	8.6	6.3
	Std. Error of Mean	2.3	0.42	3.5	2.6
	Lower 95% CI of mean	17	13	12	16
	Upper 95% CI of mean	28	15	30	29

	Biological	CONTROL	MALT	CONTROL	MALT
	Foster	CONTROL	CONTROL	MALT	MALT
	# of values	7	4	6	6
	Minimum	10	16	12	6.2
	Maximum	45	39	36	38
	Range	35	23	24	32
CD8+ CCR5	Mean	31	24	28	19
	Std. Deviation	14	10	8.9	12
	Std. Error of Mean	5.1	5.2	3.6	4.8
	Lower 95% CI of mean	18	7.4	19	6.1
	Upper 95% CI of mean	44	40	38	31
	# of values	7	4	6	6
	Minimum	23	15	30	14
	Maximum	63	65	72	60
	Range	40	50	42	46
CD8+ CXCR4	Mean	40	37	44	32
	Std. Deviation	12	25	17	19
	Std. Error of Mean	4.7	12	7	7.7
	Lower 95% CI of mean	29	-3	26	12
	Upper 95% CI of mean	52	76	62	52
	# of values	7	4	6	6
	Minimum	3.5	1.7	7	1.7
	Maximum	22	22	20	19
	Range	18	20	13	17
CD8+ HLA DR	Mean	11	11	13	9
	Std. Deviation	6.7	8.4	6	5.6
	Std. Error of Mean	2.5	4.2	2.5	2.3
	Lower 95% CI of mean	4.9	-2	6.8	3.2
	Upper 95% CI of mean	17	25	19	15
	# of values	7	4	6	6
	Minimum	8	8	9.4	11
	Maximum	20	26	17	58
	Range	12	18	7.9	47
CD8+ Ki67	Mean	12	17	14	25
	Std. Deviation	4.2	7.4	3.3	17
	Std. Error of Mean	1.6	3.7	1.3	6.8
	Lower 95% CI of mean	8.4	5	10	7.1
	Upper 95% CI of mean	16	29	17	42
	# of values	7	4	6	6
	Minimum	1	0.79	2	1.4
	Maximum	9.9	4.5	9.6	11
	Range	8.9	3.7	7.6	9.8
CD8+ PD1	Mean	4.4	2.3	4.4	4.5
	Std. Deviation	3.2	1.7	2.7	3.4
	Std. Error of Mean	1.2	0.86	1.1	1.4
	Lower 95% CI of mean	1.5	-0.4	1.5	0.93
	Upper 95% CI of mean	7.3	5.1	7.3	8.2

Table S8. CD4 Rectal Biopsy Descriptive Statistics

	Biological	CONTROL	MALT	CONTROL	MALT		Biological	CONTROL	MALT	CONTROL	MALT
	Foster	CONTROL	CONTROL	MALT	MALT		Foster	CONTROL	CONTROL	MALT	MALT
	# of values	6	4	6	6		# of values	6	4	6	6
	Minimum	22	29	29	36		Minimum	45	48	30	46
	Maximum	41	39	54	41		Maximum	83	72	89	84
	Range	20	9.4	25	4.4		Range	38	24	58	38
%CD4+	Mean	32	34	37	39	CD4+ CCR5	Mean	65	66	57	70
	Std. Deviation	7.8	4	9.2	1.6		Std. Deviation	18	12	23	14
	Std. Error of Mean	3.2	2	3.8	0.66		Std. Error of Mean	7.4	5.8	9.3	5.6
	Lower 95% CI of mean	23	27	28	37		Lower 95% CI of mean	46	47	33	56
	Upper 95% CI of mean	40	40	47	41		Upper 95% CI of mean	84	84	81	84
	# of values	6	4	6	6		# of values	6	4	6	6
	Minimum	0.053	0.29	0.81	0.27		Minimum	33	28	61	27
	Maximum	12	4.8	8.4	3.1		Maximum	65	55	73	80
	Range	12	4.6	7.6	2.8		Range	32	27	12	53
CD4+ Naïve	Mean	3.8	2.3	3.3	1.6	CD4+ CXCR4	Mean	52	47	65	55
	Std. Deviation	4.5	1.9	2.7	1		Std. Deviation	12	13	4.6	20
	Std. Error of Mean	1.8	0.95	1.1	0.41		Std. Error of Mean	4.9	6.4	1.9	8.2
	Lower 95% CI of mean	-0.92	-0.71	0.46	0.49		Lower 95% CI of mean	39	27	60	34
	Upper 95% CI of mean	8.6	5.3	6.1	2.6		Upper 95% CI of mean	64	68	70	76
	# of values	6	4	6	6		# of values	6	4	6	6
	Minimum	2.1	6	11	4.2		Minimum	0.69	2.4	16	1.1
	Maximum	26	34	22	31		Maximum	45	47	48	44
	Range	24	28	11	27		Range	44	44	31	43
CD4+ Tcm	Mean	13	17	17	15	CD4+ HLA DR	Mean	26	25	23	25
	Std. Deviation	9.6	12	4.1	9.3		Std. Deviation	15	18	12	15
	Std. Error of Mean	3.9	6	1.7	3.8		Std. Error of Mean	6.2	9.1	5	6.3
	Lower 95% CI of mean	3.2	-1.8	13	5.2		Lower 95% CI of mean	9.7	-3.8	10	8.5
	Upper 95% CI of mean	23	36	21	25		Upper 95% CI of mean	42	54	36	41
	# of values	6	4	6	6		# of values	6	4	6	6
	Minimum	61	61	62	65		Minimum	9.6	18	11	14
	Maximum	91	87	83	86		Maximum	25	41	23	20
	Range	31	27	22	20		Range	15	23	13	5.9
CD4+ Tem						CD4+ Ki67					
	Mean	77	74	75	79		Mean	16	26	15	17
	Std. Deviation	12	12	7.8	7.6		Std. Deviation	5.6	10	5	2.1
	Std. Error of Mean	5.1	6	3.2	3.1		Std. Error of Mean	2.3	5.1	2.1	0.85
	Lower 95% CI of mean	64	54	66	71		Lower 95% CI of mean	9.8	9.9	9.7	14
	Upper 95% CI of mean	90	93	83	87		Upper 95% CI of mean	22	42	20	19
	# of values	6	4	6	6		# of values	6	4	6	6
	Minimum	13	19	6.9	6.5		Minimum	4.8	1.9	4.1	2.5
	Maximum	18	27	37	23		Maximum	21	25	26	21
	Range	4.9	8	30	16		Range	16	23	22	19
CD4+ B7	Mean	16	23	16	14	CD4+ PD1	Mean	14	13	16	8.4
	Std. Deviation	1.8	3.9	11	7.1		Std. Deviation	7.3	9.4	8.1	6.8
	Std. Error of Mean	0.75	2	4.5	2.9		Std. Error of Mean	3	4.7	3.3	2.8
	Lower 95% CI of mean	14	17	4.6	6.8		Lower 95% CI of mean	6.3	-1.9	7.1	1.3
	Upper 95% CI of mean	18	30	28	22		Upper 95% CI of mean	22	28	24	16

Table S9. CD8 Rectal Biopsy Descriptive Statistics

	Biological	CONTROL	MALT	CONTROL	MALT
	Foster	CONTROL	CONTROL	MALT	MALT
	# of values	6	4	6	6
	Minimum	32	28	28	22
	Maximum	51	50	50	47
	Range	18	22	22	25
%CD8+	Mean	41	37	39	36
	Std. Deviation	6.3	9.3	10	8.6
	Std. Error of Mean	2.6	4.7	4.2	3.5
	Lower 95% CI of mean	34	22	28	27
	Upper 95% CI of mean	47	52	49	45
	# of values	6	4	5	6
	Minimum	0.11	0.04	0.098	0.07
	Maximum	3.8	3.1	2.8	1.4
	Range	3.7	3	2.7	1.4
CD8+ Naïve	Mean	0.92	1.2	1.1	0.6
	Std. Deviation	1.4	1.3	1	0.46
	Std. Error of Mean	0.58	0.65	0.45	0.19
	Lower 95% CI of mean	-0.57	-0.82	-0.18	0.12
	Upper 95% CI of mean	2.4	3.3	2.3	1.1
	# of values	6	4	5	6
	Minimum	1.1	2.5	3	1.2
	Maximum	17	15	6.5	6.7
	Range	16	12	3.4	5.5
CD8+ Tcm	Mean	5.2	8.8	4.5	4.9
	Std. Deviation	5.9	6.2	1.4	2.1
	Std. Error of Mean	2.4	3.1	0.64	0.86
	Lower 95% CI of mean	-0.94	-1.1	2.7	2.6
	Upper 95% CI of mean	11	19	6.3	7.1
	# of values	6	4	5	6
	Minimum	61	71	85	79
	Maximum	96	97	92	95
	Range	35	26	6.6	17
CD8+ Tem	Mean	87	83	89	89
	Std. Deviation	13	11	2.4	5.8
	Std. Error of Mean	5.4	5.3	1.1	2.4
	Lower 95% CI of mean	73	66	86	83
	Upper 95% CI of mean	101	100	91	95
	# of values	6	4	5	6
	Minimum	72	70	76	45
	Maximum	88	88	93	87
	Range	17	18	18	43
CD8+ B7	Mean	83	82	83	76
	Std. Deviation	6.9	8.4	7.6	16
	Std. Error of Mean	2.8	4.2	3.4	6.5
	Lower 95% CI of mean	76	69	74	59
	Upper 95% CI of mean	91	96	93	93

	Biological	CONTROL	MALT	CONTROL	MALT
	Foster	CONTROL	CONTROL	MALT	MALT
	# of values	6	4	5	6
	Minimum	21	30	24	24
	Maximum	86	90	74	97
	Range	64	60	50	72
CD8+ CCR5	Mean	53	62	45	60
	Std. Deviation	28	30	18	30
	Std. Error of Mean	11	15	8.1	12
	Lower 95% CI of mean	24	15	22	28
	Upper 95% CI of mean	83	110	67	91
	# of values	6	4	5	6
	Minimum	13	22	31	21
	Maximum	44	45	55	48
	Range	31	23	24	27
CD8+ CXCR4	Mean	31	36	43	39
	Std. Deviation	13	11	10	9.7
	Std. Error of Mean	5.1	5.4	4.5	4
	Lower 95% CI of mean	18	19	30	28
	Upper 95% CI of mean	44	53	55	49
	# of values	6	4	5	6
	Minimum	2.3	1	8	0.94
	Maximum	36	35	36	44
	Range	33	34	28	43
CD8+ HLA DR	Mean	18	19	18	22
	Std. Deviation	11	14	12	15
	Std. Error of Mean	4.7	7	5.1	6.2
	Lower 95% CI of mean	6	-3.6	3.5	5.9
	Upper 95% CI of mean	30	41	32	38
	# of values	6	4	5	6
	Minimum	16	24	9.5	14
	Maximum	25	68	39	45
	Range	8.6	44	30	31
CD8+ Ki67	Mean	21	38	21	27
	Std. Deviation	3.5	20	12	14
	Std. Error of Mean	1.4	10	5.2	5.5
	Lower 95% CI of mean	17	5.5	6.6	13
	Upper 95% CI of mean	25	70	35	42
	# of values	6	4	5	6
	Minimum	2.3	0.44	1	0.21
	Maximum	8.6	6	4.7	11
	Range	6.2	5.5	3.6	10
CD8+ PD1	Mean	4.8	3.3	2.8	3.3
	Std. Deviation	2.2	2.9	1.5	4
	Std. Error of Mean	0.91	1.5	0.65	1.6
	Lower 95% CI of mean	2.4	-1.4	1	-0.85
	Upper 95% CI of mean	7.1	7.9	4.6	7.5
